# Lack of Benefit on Treating Asymptomatic Bacteriuria Prior to Cardiovascular Surgery: a Systematic Review and Meta-Analysis

**DOI:** 10.21470/1678-9741-2018-0276

**Published:** 2018

**Authors:** Sergio Alejandro Gómez-Ochoa, Blanca Beatriz Espín-Chico

**Affiliations:** 1 Researcher GERMINA-UIS Group. School of Medicine, Health Sciences Faculty, Universidad Industrial de Santander, Bucaramanga, Colombia.; 2 Public Health Faculty. Escuela Politécnica Superior de Chimborazo, Riobamba, Ecuador.

Dear Editor,

Asymptomatic bacteriuria (ASB) represents a relatively common finding among preoperative patients, being a significant cause of delays and costs for health institutions, mainly due to the perceived risk of hematogenous spread and surgical site infection (SSI) as it leads to surgical reprogramming and antibiotic treatment^[1,2]^. However, recent evidence has been showing that in most of the scenarios ASB do not increase the risk of any infectious complications by itself, as in the case of joint replacement^[3,4]^. However, in the cardiovascular surgery context there is few evidence about the impact of ASB in the SSI rate, this last has a reported incidence of 1%-10% and confers affected individuals a high risk of mortality, which can reach up to 25% in cases of deep SSI^[5,6]^. Because of this, there is a substantial need of evaluating the potential role of ASB, in order to create optimal recommendations for the SSI prevention in these patients. This systematic review and meta-analysis was performed to evaluate the benefit of treating ASB prior to cardiovascular surgery regarding the risk of SSI.

The present systematic review and meta-analysis was conducted in accordance with the Cochrane Handbook for meta-analyses and systematic reviews and the PRISMA (Preferred Reporting Items for Systematic Reviews and Meta-analyses) Statement. The literature search was performed by two authors using the databases Medline, Embase, PubMed, EBSCOHost, SciELO, LILACS and the Cochrane Central Register of Controlled Trials using the following terms: asymptomatic bacteriuria; bacteriuria; nitrites; pyuria; asymptomatic leukocyturia; urine analysis; urinalysis; cardiac surgery; heart surgery; cardiovascular surgery; cardiothoracic surgery. Eligible studies corresponded to randomized controlled trials, cohorts and case-control studies that evaluated the benefit of treating ASB in patients taken to cardiovascular surgery. Also, the studies included had to be written in in English, Spanish or Portuguese, published after January 1^st^ of 1970 and had to be available on full text. The reviewers evaluated the title at first, excluding those without any correlation with the objective of the study, then the abstracts were reviewed, removing the duplicates, finally the suitable articles were selected for full text review. For outcomes with suitable data for combination, results were meta-analyzed using Review Manager 5.3 (Nordic Cochrane Center, Cochrane Collaboration 2009, Copenhagen, Denmark).

**Fig. 1:**
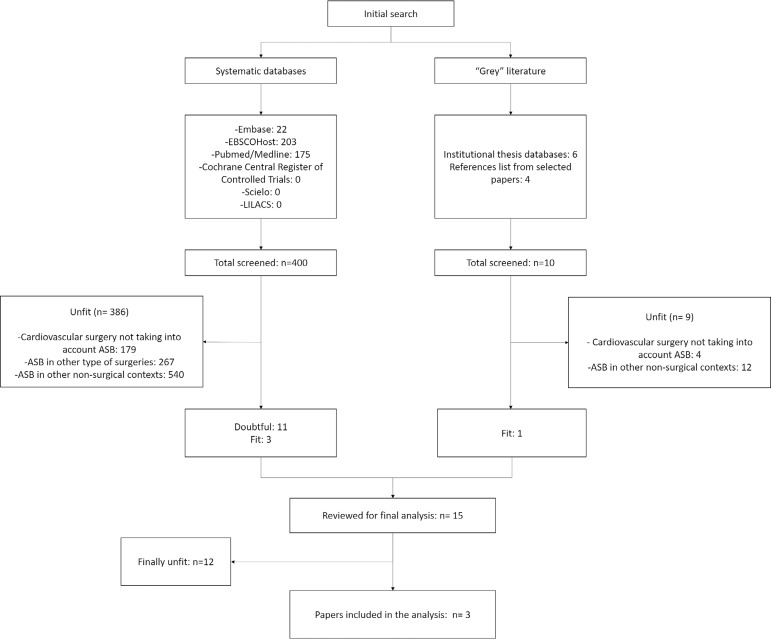
Flowchart of included studies.

**Fig. 2:**
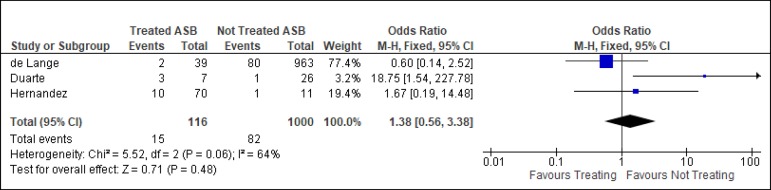
Forrest plot of the risk of surgical site infection (SSI) in patients taken to cardiovascular surgery with treated vs untreated asymptomatic bacteriuria (ASB).

After the initial search, 410 articles were identified, being 407 excluded for not being related to the study's objective (Figure 1). Three studies involving a total of 1116 patients were included, being the individuals mainly males (47.4%), with a mean age at the procedure of 67.4 years. The procedures performed corresponded to non-valvular coronary artery bypass grafting (CABG) in 473 (42%) patients, valvular replacements in 567 (51%) cases and thoracic aortic surgeries in 76 (7%) patients. Regarding the treatment, 116 patients had specific antimicrobial treatment before the surgical procedure, having these patients a higher rate of SSI when compared to the ones not treated (12.9% *vs*. 8.2%), however, this result did not achieve a statistically significant value (*P*=0.086). To compliment this finding, a random effects model meta-analysis was performed to evaluate the benefit that treating bacteriuria may confer regarding SSI risk. A moderate heterogeneity was observed (I_2_=64%) among the included studies, encountering a not significant effect of the antimicrobial intervention over the infectious complications chosen (OR 1.38; 95% CI 0.56, 3.38) (Figure 2).

As for other kind of surgical procedures, our meta-analysis results suggest that ASB treatment may not influence the development of SSI in included types of cardiac and cardiovascular surgeries, therefore active screening for this condition may not be useful, as the only goal of identifying ASB in the clinical practice is to treat it. In consequence, neither preoperative urine culture or ASB treatment could be recommended, as they may represent an inappropriate use of resources without a clear benefit. On the other hand, they may expose the patients to prolonged hospitalizations and unjustifiably to antibiotics with multiple adverse effects, as allergic reactions, post antibiotic diarrhea and resistance induction, also increasing health care costs^[7,8]^.

Significant limitations appear when analyzing this study, as the very low number of available studies and the retrospective and non-randomized design of the included ones makes impossible to generate a strong recommendation, giving for now only a view of the trend of the research performed in this area. Larger multicenter studies are needed for establishing the role of asymptomatic urinary tract colonization in infectious complications of surgical procedures as cardiovascular surgery.
